# The Techno-Functionality of Chia Seed and Its Fractions as Ingredients for Meat Analogs

**DOI:** 10.3390/molecules29020440

**Published:** 2024-01-16

**Authors:** Caroline Senna, Luiza Soares, Mariana Buranelo Egea, Sibele Santos Fernandes

**Affiliations:** 1 School of Chemistry and Food, Federal University of Rio Grande, Av Italy km 8, Carreiros, Rio Grande 96203-900, Brazil; carolalmeidasenna@gmail.com (C.S.); luizasribeiro94@gmail.com (L.S.); 2 Goiano Federal Institute, Campus Rio Verde, Km 01, Rural Area, Rio Verde 75901-970, Brazil

**Keywords:** *Salvia hispanica*, meat-like products, coproducts, mucilage, defatted chia flour

## Abstract

Eating practices are changing due to awareness about meat consumption associated with social, ethical, environmental, and nutritional issues. Plant-based meat analogs are alternatives to conventional meat products that attempt to mimic all the inherent characteristics of meat fully. Therefore, the search for raw materials that provide these characteristics is increasing. Chia seeds have excellent potential as a functional ingredient in these products since they are a source of proteins, lipids, and fibers. Allied with this, the full use of chia through the seed and its fractions highlights the numerous beneficial characteristics of the formulation regarding nutritional characteristics and techno-functionality. Therefore, this review aims to highlight the potential of chia seed and its fractions for applications in meat-like products. Chia seeds are protein sources. Chia oil is rich in polyunsaturated fatty acids, and its application in emulsions ensures the oil’s nutritional quality and maintains its technological characteristics. Defatted chia flour has a high protein content and can be used to extract chia mucilage. Due to its high emulsification capacity, chia mucilage is an effective ingredient for meat products and, consequently, meat-like products. Therefore, this literature review demonstrates the strategic potential of using chia seeds and their fractions to develop meat analogs.

## 1. Introduction

Demand for meat-like products has increased worldwide because more consumers are looking for similar alternatives to meat products [1]. The increase in the consumption of and demand for meat-like products stems from dietary changes aimed at environmental sustainability and animal ethics and are associated with improved human health and the prevention of various diseases, such as cancer, cardiovascular disease, diabetes, hypertension, and obesity [2].

On the market, most meat-like products are soy-based and contain other ingredients, mostly food additives, to imitate the texture, color, flavor, and inherent characteristics of meat [2]. Other raw materials are peas, lentils, chickpeas, quinoa, buckwheat, wheat gluten, rice, sorghum, potatoes, and oilseeds [3].

However, available meat analog products still have deficiencies in their characteristics because combining all the attributes of meat in a product using vegetable materials is difficult. Therefore, alternative sources of raw materials have been studied, alone or in combination with a proposal to develop meat analog products. A potential raw material is chia seed and its fractions, such as oil, whole flour, defatted flour, and polysaccharide-rich gel (mucilage).

Chia seeds contain up to 25% protein; 33% oil, of which almost 70% is omega 3 polyunsaturated fatty acids; and 6% chia mucilage [4,5,6]. By 2027, it is believed that the chia seed market will grow by 6.5% per year, and this impact will be more significant in Europe, which has a large number of consumers concerned about consuming healthier foods that have less impact on the environment, such as meat analogs [7].

Therefore, this review presents chia seed and its fractions as a strategy for developing meat-like products. It highlights the techno-functional characteristics of this seed, making it an excellent candidate for ingredients in meat-like products.

## 2. Meat Analogous Products

The market for replacing animal protein with vegetable protein is driven by concerns about the environmental impact of consumers, many of whom are interested in being more sustainable, and the idea that plant-based food can be healthier. Regardless of the reason, the main discussion held is that it is necessary to diversify protein sources, mainly due to their seasonality, place of production, cost, and price for the final consumer. Furthermore, as each protein source has a specific amino acid profile and digestibility and bioavailability characteristics, the consumer must diversify their consumption to meet nutritional needs [8,9,10].

As a result of this consumer need that has been growing considerably, the food industry has been developing products that are “meat substitutes”, “meat-like products”, “meat analogs”, or “plant-based”. According to Innova Market Insights (IMI), there was a 59% growth in plant-based product launches in August 2021 compared to 2020, which is a result of the demand of different consumers (vegetarians, vegans, and flexitarians) for greater variety in their diet [11].

Meat analogs are plant-based products that contain alternative proteins—protein from a vegetable source—that aim to mimic genuine meat’s fibrous appearance, texture, flavor, color, and nutritional properties [3,12]. There are several forms of meat-like products that are symbolic of meat, such as hamburgers, chicken strips, sausages, cured meat, fermented products, dried meat, and beef jerky, that have an appearance, structure, and flavor similar to meat and reminiscent of the sensations of meat consumption [13]. The industry has sought to develop these types of products, and the ground category (hamburgers and chicken nuggets), muscular type (chicken meat or cuts similar to steaks), and products in the emulsions category (sausages) are among the most produced [3].

In most countries, the food industry has faced a significant problem: regulatory issues regarding these new products. This aspect has advanced a lot recently. For example, after jointly founding the Alternative Proteins Council (a communication channel for the sector), New Zealand and Australia released on 24 June 2022 an industrial guideline for labeling meat-alternative products, to come into force in the following 24 months [14]. Although these are the only guidelines to date in the world for this sector, the biggest concern appears to be related to conscious consumption. Therefore, according to these guidelines, labeling must make it clear to the consumer that these products do not contain any animal derivative. In addition, labeling cannot contain indiscriminate claims related to health or the demonization of animal protein consumption.

Meat-like products aim to provide the consumer with all the biological, nutritional, and sensory characteristics of meat. Thus, characteristics linked to these products, such as flavor, color, texture, and sensory properties, are requirements for preparing these products when the base is plant. Furthermore, these attributes are considered as relationships between references and affective memory when consuming products of animal origin [15].

To provide acceptable sensory characteristics similar to those of animal meat, a plant-based meat analog product is generally composed of 50–80% water, 4–25% protein, 2–30% carbohydrates, 0–15% lipids, and 0–15% additives, including binding agents, seasonings, flavorings, salt, and pigments [16]. Many other embedded, fermented, salted, and cooked products are obtained from meat, such as hamburgers, meatballs, sausages, steaks, ground beef, and chicken nuggets. Among these, the hamburger is the most studied restructured product with the most excellent commercial variety [17]. Chen et al. [18] evaluated different commercially available meat analog products and found that plant-based beef hamburgers were more popular among consumers compared to other meat-alternative products.

Alternative proteins often sourced from cereals, legumes, oilseeds, and fungi work to form gel networks that can act as the backbone of meat-like fibrous structures, which are very important and challenging to imitate to achieve characteristics similar to those of meat products [18,19]. In this sense, soy seed is the ingredient that stands out for the protein portion because soy protein demonstrates a greater availability of essential amino acids when processed to provide texture [20,21]. However, in recent years, the market for plant-based products has been expanding because of social demands. In addition, the search for and innovation of alternative legume protein sources has been growing to aid the improvement and quality of the products offered on the market. Therefore, cereal proteins (wheat, rice, barley, and oats) and legume proteins (beans, peas, and lentils, among others) have also been researched as alternative sources of protein [16]. Protein isolates and concentrates from oats, peas, wheat, and sunflower are examples of fibrous sources for meat analogs [22,23].

Lipids are responsible for the juiciness, tenderness, smoothness, flavor, nutritional value, and loss of storage stability of plant-based meat analog products [24]. However, the lipids used are derived from plants, which are entirely different from animal fats in terms of chemical composition, degree of unsaturation, chain length, isomerization, crystalline forms, and physicochemical properties. The vegetable oils used for meat analogs originate from canola, coconut, sunflower, corn, rapeseed, and linseed [18].

For these reasons, research and development for meat analogs still have a long way to go in both academia and industry to seek plant sources that faithfully embody and imitate meat. While there are still challenges in processing these products, some plant sources, such as chia seeds and their fractions, have the potential to supply these characteristics.

## 3. Chia Seed

*Salvia hispanica* L., also known as chia, is a herbaceous plant belonging to the Plantae kingdom, Lamiaceae family, *Salvia* genus, and *Hispanic* species [25]. This seed is grown commercially in Mexico, Guatemala, Bolivia, Australia, Peru, Argentina, America, and Europe [26,27]. Of the 900 seed species of the Lamiaceae family, 61 are commercially cultivated in Brazil [28].

The plant is cultivated mainly for the use of its seeds, which are small, oval, white and black seeds measuring less than approximately 1 mm thick; therefore, when flowering, seeds also present these colors [25,29].

In Brazil, chia is an exotic plant that has been increasing its importance and production in recent years, being planted mainly in the western regions of Paraná and northwest of Rio Grande do Sul [30,31], as well as in Mato Grosso do Sul [32]. In the other states of the country, chia does not have a significant area for planting. However, the conditions in Brazil regarding temperature, altitude, and precipitation are compatible with the conditions required by the culture, thus there is the possibility of expansion [31]. Climatic conditions, geographic location, nutrients, temperature, soil conditions, and year of cultivation affect chia seeds’ chemical composition and nutritional value [33,34,35]. 

Compared to other oilseeds, such as quinoa, flax, and sunflower, the chemical composition of chia seeds shows their high nutritional value [36]. Chia seeds have high values of lipids, carbohydrates, and proteins [4,7,33]. Chia seeds have a high oil content, about 28 to 33%, and are rich in polyunsaturated fatty acids, mainly omega 3 (linolenic acid, 57 to 69%) and omega 6 (linoleic acid, 17 to 22%) [4,36,37]. In smaller quantities, palmitic, stearic, and oleic acids are also found [37]. Several studies point to the use of chia seeds due to the high percentage of fatty acids present, which are important for overall health and antioxidant and antimicrobial activities [38,39]. 

Chia’s fiber content also makes it a good candidate for use in producing functional foods. Chia seed has a high dietary fiber content, around 35.5%, which allows for a lower glycemic index and can provide water and oil retention capacities to food formulations [40]. The fiber is of two types: soluble, more commonly known as chia mucilage, and insoluble [29]. 

Chia seeds are also considered a source of phenolic compounds (350 mg/100 g of seed) with antioxidant capacities (15.5% inhibition) and of other biocompounds such as beta-carotene, tocopherol, chlorogenic acid, and caffeic acid (1220 mg/100 g of seed) [38]. The most reported flavonoids for chia seeds are myricetin, quercetin, kaempferol, and synaptic acid [31,41,42].

**Figure 1:**
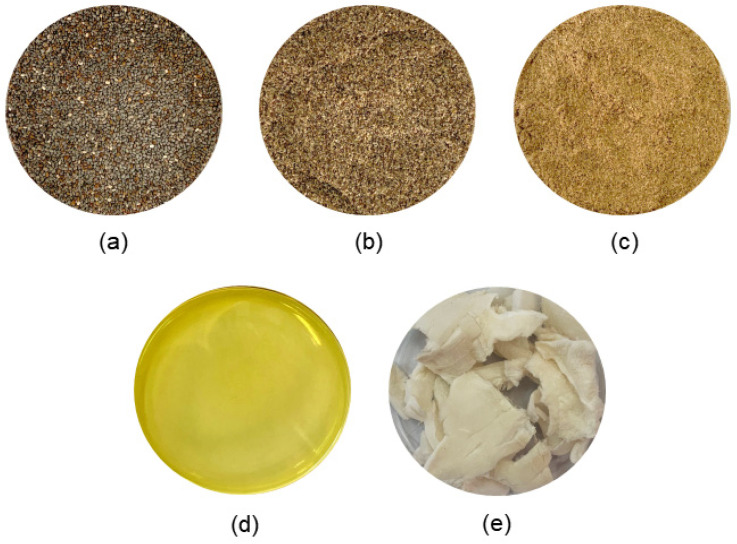
Chia seed (**a**), whole chia flour (**b**), partially defatted chia flour (**c**), chia oil (**d**), and freeze-dried chia mucilage (**e**).

From chia seeds, different products and fractions can be obtained, using seeds whole, ground, or soaked in water in the form of flour, oil, or gel (mucilage) [43,44]. Figure 1 shows the chia seed (a) and its coproducts. These products can have the same characteristics that their raw material presents, such as antioxidant properties, functional properties, and the ability to form emulsions and absorb water. Consequently, studies have been carried out using chia seeds or their by-products as an ingredient in the food industry, where the seeds can be used in different formats [43,44].

## 4. Chia Ingredients

**Figure 2:**
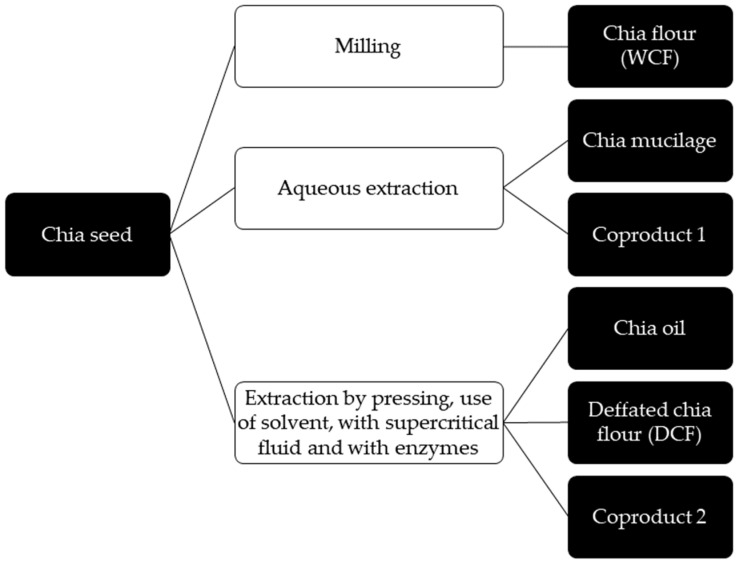
Products and coproducts obtained from chia seed.

Chia seed has become one of the most recognized seeds because of its nutritional and functional properties [38] as well as its wide application in plant-based products using chia products that can be obtained from chia seed hydration (chia mucilage), chia flour, and chia oil or the extraction of biocomponents of any of its fractions or by-products [43]. Figure 2 shows all the products and fractions that can be obtained from chia seeds that have massive potential for applications in developing meat analogs.

### 4.1. Whole Chia Flour (WCF)

Whole chia flour (WCF) contains the same composition as the original chia seed since chia flour is obtained directly by grinding the chia seed [45]. This chemical composition of WCF may change according to the origin of the seed, soil quality, and climate. WCF has a high capacity to retain and absorb water and can be used as an emulsifying agent, thickener, stabilizer, or antifreeze agent in the food industry [46]. It can also be used for its gelling and water-binding capacities as well as its microstructure and texture in foods [45]. Due to these characteristics associated with protein content, WCF is mainly used in bread [47], pound cakes [48], and gluten-free premixes [46]. Some studies have used WCF in meat products due mainly to emulsifying, gelling, and/or stabilizing properties; however, it is not used in meat-like products [49,50,51].

Pintado et al. [51] developed emulsion gels prepared with olive oil, WCF, and cold gelling agents (transglutaminase, alginate, or gelatin) for use as fat replacers in reduced-fat frankfurter sausage formulations. The developed frankfurter sausages had a lower saturated fatty acid content, higher mono- and polyunsaturated fatty acid contents (400% increase in omega 3), good fat and water-binding properties, and reduced hardness compared with controls. Sensory properties (color, flavor, texture, and general acceptability) were not affected by the incorporation of emulsion gels, and all frankfurter sausages were considered acceptable.

Similarly, Paglarini et al. [49] used WCF (2.5%) as a gelling agent to formulate soybean oil emulsion gels to replace 10% and 20% of pork back fat in Bologna sausages. Replacing 20% of pork fat with a WCF emulsion resulted in Bologna sausages with a 30% reduction in total lipid content, a higher omega 3 content, reduced hardness, and a 10% reduction in protein content compared with controls. Regarding the microstructure of the Bologna sausages, adding WCF affected the protein matrix, resulting in a more compact structure. This behavior was due to the composition of the flour, which is rich in proteins and fibers and has an excellent binding capacity with water and fat and thus may explain these results.

Pires et al. [49] created Bologna sausages by replacing pork back fat with Echium oil and partially replacing lean beef with 10% WCF. The sausages produced showed an increase in lipid content, from 12.33 to 17.94 g/100 g, and a consequent improvement in the lipid profile of the meat product compared with controls. However, the sausages were not well accepted through sensory analysis, with aroma being the most negative parameter.

Therefore, it is clear that WCF acting as a gelling agent can be used as an ingredient to develop meat analogs, probably due to the chia mucilage in this flour.

### 4.2. Defatted Chia Flour (DCF)

Defatted chia flour (DCF) is a coproduct obtained through the extraction of chia oil by methods such as pressing, solvent extraction, and supercritical fluids [4]. However, DCF that is commercially available for consumption is obtained by cold pressing the chia seed to remove the crude oil [52], and it has been commercialized at a low cost for use as animal feed. Due to its high yield, the amount of DCF produced is very high. For every 1000 kg of chia seed used for oil extraction, about 650 to 700 kg of DCF is generated, which is higher than the demand for its use in animal feed [18]. Therefore, other alternatives for DCF are an area of research that is gaining prominence as a strategy to add value to this fraction, taking advantage of its excellent nutritional composition [53]. 

The chia oil extraction process using the pressing method is not completely efficient as it cannot remove the oil intrinsically pressed into the seed structure, which is associated with the difficult maintenance of operational conditions, such as temperature and humidity. Therefore, commercial DCF is partially defatted chia flour (PDCF) as there is still a significant lipid content in this flour. Thus, compared to whole flour, PDCF has a reduction of 80% in total lipid content, resulting in a remaining lipid content of 5.0 to 6.5% [7,52]. This reduction results in an increase in other components that are present, such as proteins and fibers [54].

The protein content in PDCF is around 47% higher than in WCF [52]. In PDCF, the amino acids present in major contents are glutamate (18.46%), arginine (12.69%), aspartate (9.72%), and serine (7.40%). Of the amino acid profile, 33.7% corresponds to essential amino acids (histidine, threonine, valine, lysine, isoleucine, leucine, phenylalanine, methionine, and tryptophan) [53]. In other words, these amino acids can be obtained by including PDCF in the diet.

When proteins are extracted from DCF via alkaline extraction and precipitation at the isoelectric point followed by enzymatic hydrolysis, there is a 3.8% increase in the peptide content. In addition, the protein concentrate obtained from PDCF has a higher antioxidant capacity than whole flour [53]. 

The PDCF fraction also has a relevant fiber content. Comparing PDCF with WCF, the total dietary fiber contents are 48% and 38%, respectively; insoluble fiber contents are 39% and 35% and soluble fiber contents are 9% and 3%, respectively [7].

Another relatively important point is the presence of polyphenols in PDCF. Polyphenols are a group of compounds that have antioxidant properties. This antioxidant capacity depends on the polyphenol content and chemical structure [55]. Mas et al. [56] related thirteen compounds to DCF that were identified as hydroxycinnamic acids and structurally related to caffeic acid, and nearly 60% of these compounds contained polyphenols, rosmarinic acid, and fertaric acid. Ten flavonoids, including quercetin, kaempferol, myricetin, and some glycosylated derivatives, were also found.

As with WCF, PDCF has considerable protein and fiber content and antioxidant components [52,53,57]. Thus, PDCF appears to be an interesting ingredient for different foods, such as meat analogs, as it generates supplemented products, is rich in natural antioxidants, and reduces industrial waste [58]. However, despite the excellent properties associated with this chia coproduct, there are still no studies in the literature on the use of PDCF in meat analogs. 

### 4.3. Chia Mucilage

Chia mucilage (approximately 5.8% of the seed) is completely exuded from the seed when it encounters water at room temperature for approximately 2 h. The exudate gel is strongly attached to the seed and is separated from the seed by mechanical forces. Afterwards, it is dried in an oven at 50 °C or by freeze-drying for later use [5]. When the gel formed from the chia seed does not separate, it is called chia gel, and when the gel formed from the chia seed is separated, it is called chia mucilage.

Chia mucilage has a favorable nutritional value for various applications due to the concentration of monosaccharides from the chia seed, which is what gives it its excellent technological properties. Chia mucilage is a polysaccharide with structural units described as a tetrasaccharide, with a main chain composed of (1→4)-β-D-xylopyranosyl-(1→4)-α-D-glucopyranosyl-(1→4)-β-D-xylopryanosyl units with a 4-O-methyl-α-D-glucuronic acid branch [59]. 

Various monosaccharides are obtained from the hydrolysis of chia mucilage, with arabinose and xylose being the main ones, representing approximately 85% of the total [6]. In smaller amounts, the monosaccharides glucose, fructose, galactose, rhamnose, and mannose are also found [60]. In addition to these components, chia mucilage contains 8% protein, which aids emulsifying properties, and 1% oil [5].

Chia mucilage presents properties such as water retention and absorption capacity and can be used as an emulsifying agent, emulsion stabilizer, foam stabilizer, suspending agent, or binding agent, and can also act as a fat substitute as it can hydrate and develop viscosity [5,7]. The properties of the formed gel confer characteristics and qualities when applied to different products in the food industry, thus improving sensory characteristics, such as the texture and nutritional value of food products [59]. In addition, the rheological behavior of chia mucilage demonstrates that its mechanical properties are resistant to heat treatment, which is extensively used in the food industry [61].

Some polysaccharide-based ingredients, such as mucilage and by-products obtained from chia seeds, are used to provide characteristics that simulate sensory and physical–chemical attributes and can be used as water retainers, emulsifiers, thickeners, stabilizers, and gelatinizers of conventional products [62,63,64]. Despite the wide application of chia mucilage, few studies still use it in meat-like products.

Câmara et al. [61] evaluated the use of chia mucilage as a functional ingredient in emulsified meat model systems, replacing 50% of the fat content with chia mucilage. As emulsion stability and water loss are relevant quality parameters for meat products, the authors found significant improvements in the stability of meat emulsions and decreased water exudation in sliced products with chia mucilage. In addition, there was an increase in hardness (>35%), but the authors believed that changes in the formulations could overcome this inconvenience.

In addition to replacing fat in meat products, chia mucilage can be used as an additive to replace added phosphates. Câmara et al. [65] concluded that chia mucilage (powder and gel) could substitute phosphates in low-fat Bologna sausages. The authors tested different treatments: a control treatment with the addition of 0.5% phosphate; four treatments without phosphates and with the addition of 2% and 4% chia mucilage powder and the addition of 2% and 4% chia mucilage gel; and two treatments with the addition of 0.25% phosphate and 2% and 4% chia mucilage gel. Most samples with mucilage were less firm and less chewy. Among the treatments, the one with 2% chia mucilage gel proved to be a viable strategy to replace 50% of phosphates in low-fat Bologna sausages due to its functional properties.

Liu et al. [66] created beef patties by replacing pork fat (20%, 40%, 60%, 80%, and 100%) with emulsion gels prepared with chia mucilage and olive oil. Compared to the control group, the beef patties developed had reduced fat and protein contents and greater lipid stability. Additionally, polyunsaturated fatty acid levels were increased by up to 36% (100% fat replacement levels).

From the previously mentioned works that used chia mucilage as an ingredient in meat products, its potential as a healthier ingredient has become evident. Thus, its use in meat-like products presents a gap to be filled, with possible satisfactory results.

### 4.4. Chia Oil

The sensory properties, mainly those of tenderness and juiciness, that are different from meat products are one of the main limitations of plant-based meat analogs [67]. Thus, the source of fat used in meat-like products is of great technological, sensory, and nutritional importance [68]. As a substitute for animal fat, vegetable oils with several lipid profiles, such as sunflower, canola, palm, coconut, and chia, among others, have also been studied [69]. 

Chia oil represents one of the primary uses of chia seed by-products [70]. Different solvents (ethyl acetate, acetone, propane, petroleum ether, and hexane) and extraction techniques (cold and hot pressing, Soxhlet, ultrasound-assisted extraction, and pressurized liquid extraction) are applied to obtain chia seed oil [71,72]. Because it is a method that preserves natural antioxidants and is associated with the growing demand for more natural products, extraction by cold pressing is the most used method of obtaining chia oil, which is used for the disposal of oil in the market [7].

It is known that most meat-like products use soy as the base raw material. The efficiency of using vegetable oils to improve the juiciness and odor of soybeans has already been proven [73]. In addition, they help in the darkening of the product when cooked [74]. Although there are many products on the market and many studies on the development, properties, and characterization of meat-like products, the use of chia oil applied to these products is still nonexistent. Different studies point to the use of chia oil in bakery products, cookies, and pasta [75,76,77].

Carvalho et al. [78] developed lamb patties that replaced animal fat with chia oil with the addition of guarana seed, pitanga leaf extracts, and natural antioxidants. The fatty acid profile of the lamb burgers was improved by replacing the fat with chia oil emulsion. The extracts delayed the burgers’ discoloration, giving a greater red color intensity and delaying lipid and protein oxidation throughout storage time.

Another way of using vegetable oils in plant-based meat is through emulsions, which promote an increase in the oil content in high-moisture extrudates and improve texture properties, better imitating real meat. Chia oil emulsions were prepared using multilayers obtained by layer-by-layer electrostatic deposition technology [79], gelled emulsions [80,81,82], and hydrogelled emulsions [83]. Heck et al. [83] found that the hydrogelled emulsions of chia oil and linseed oil could retain practically all the oil (25%) used in preparing the emulsion.

To further improve the characteristics of the emulsion, Botella-Martinez [80] evaluated the use of adding amaranth flour to a gelled emulsion of chia or hemp oil as a fat substitute in beef burgers. Adding gelled emulsion decreased the fat content in the burgers by between 12% and 33%. In addition, in the amaranth emulsion, there was a decrease in the amount of palmitic, stearic, and oleic fatty acids and an increase in the amount of linolenic fatty acids (γ-linolenic and α-linolenic acid); in the amaranth–chia emulsion, there was an increase in α-linolenic acid. 

Botella-Martínez et al. [81] developed vegetable burgers using gelled emulsions based on chia and hemp oil as a source of fat and beet juice as a coloring ingredient. The authors obtained burgers with a low fat content (<3%) and notable protein (18.6–19.5%) and dietary fiber (14.5–16.2%) contents. Sensorially, the vegetable burgers were classified as having good general acceptability, with varying scores from 4 to 7 for color, overall appearance, texture, and flavor.

Lucas-Gonzalez et al. [82] replaced 5% and 10% of a fat replacer in pork burgers with chia oil emulsion with the addition of chestnut flour, gellan gum, and water. The emulsions were considered adequate. They improved cooking performance and increased by more than a thousand times linolenic and linolenic acid contents compared with controls. The 10% emulsion burger obtained higher sensory scores. 

Heck et al. [83] replaced 20%, 40%, 60%, 80%, and 100% of pork back fat with hydrogelled emulsion containing 12.5% chia oil, 12.5% linseed oil, 70% water, 1% polysorbate 80, and 4% kappa carrageenan. Replacing pork fat with the hydrogelled emulsion did not negatively affect the technological properties of the burgers. However, there was an increase in the hardness and chewiness of the samples due to the increase in the protein: lipid ratio. The smallest substitution evaluated (20%) provided a significant three-fold increase in the omega-3 content in the cooked sample and the largest substitution evaluated (100%) provided a seven-fold increase.

Given this, the studies mentioned above that used chia oil emulsions to replace fat in meat products show that this could be a promising strategy to produce meat analogs with a healthier lipid profile and good technological characteristics.

### 4.5. Coproduct 1 and Coproduct 2

Chia seed can reach 25% protein; it has a higher protein concentration compared to other traditional grains, such as wheat (14%), barley (9.2%), oats (15.3%), corn (14%), and rice (8.5%) [84]. Environmental and agronomic factors affect the amount of protein in chia seeds [35]. This protein content is concentrated in coproducts 1 and 2 (Figure 1) due to the extraction processes of the other components. Thus, excellent protein components are to be used in food production.

As previously seen, chia mucilage has reduced levels of protein and oil as well as of linolenic and linoleic acids compared to the contents present in the seeds, indicating that protein and oil are retained in the cellular structure and not extracted by the mucilage [5]. This means that coproduct 1 has high lipid and protein contents and can also be used for oil extraction and to obtain protein concentrates and isolates. It can also be used to provide different functions (enhancing nutritional profiles and assisting in technological properties) with a single ingredient.

Chia proteins can be concentrated through protein-rich fractions that have a protein content of less than 65% and are obtained simply by physical methods (sieving), concentrates with a protein content of 65 to 90%, and isolates with a protein content greater than 90%, using chia products (flour and defatted flour) as well as coproducts [85]. In addition, from the hydrolysis of chia proteins, it is possible to obtain bioactive peptides with promising biofunctional properties, such as hypoglycemic, hypolipidemic, hypotensive, anti-inebriation, antithrombotic, antihypertensive, antimicrobial, antibiofilm, and antioxidant bioactivities [18,86,87].

The protein fractions in the highest concentrations in chia are albumin and globulin types, followed by prolamin and glutelin [26]. All essential amino acids for human nutrition are present in chia seeds, such as leucine (dominant), isoleucine, lysine, methionine, phenylalanine, threonine, tryptophan, histidine, and valine. In the case of non-essential amino acids, glutamic acid is the most dominant. When evaluating chia protein concentrates, they have about 38.7% of essential amino acids and 61.3% of non-essential amino acids. In contrast, hydrolysates have more non-essential amino acids, with 81.2% and 18.8% of essential amino acids [86]. This variation in the composition of essential and non-essential amino acids in concentrates and hydrolysates is due to hydrolysis conditions, such as the enzyme used, type of method, way of breaking the peptide bonds, breaking speed, and solubility [88,89].

In addition to the high protein content that can help in the nutritional profile of foods and the peptides that can confer some bioactivity, the proteins present in chia can also be used as a food ingredient due to their functional properties, using emulsifying, foaming, and gelling properties as examples that are widely used in the food industry to obtain products such as mayonnaise, desserts, meat, and some beverages [90]. In the literature, works related to the composition and extraction of amino acids and the study of technology and functionality, such as foaming and water/oil retention capacities of chia seed proteins, are presented [25,91], as well as studies on the functional properties of chia proteins and their peptides [26,92].

Regarding the application of chia proteins in food, studies are focused on synergistically obtaining a product with a higher protein content and some bioactivity, mainly antioxidant capacity, with improved technological properties. Madruga et al. [93] evaluated the content of hydrolyzed chia proteins and their technological, sensory, and antioxidant characteristics for wheat and rice bread. The authors obtained bread with good properties, including increased volume, softness, and antioxidant activity. Sensorially, the characteristics were maintained by adding up to 3 mg of chia hydrolysate/g of flour. In addition, it is known that rice-based products (gluten-free) lack technological properties, such as specific volume and texture, which have now been improved, approaching characteristics similar to those of traditional wheat-based bread due to the addition of chia hydrolysate.

Seed germination, which aims to intensify the protein content of chia seeds, has been used as an alternative for food products. Ghafoor et al. [42] obtained an increase of only 2% of the protein content in germinated chia seeds. However, other compounds, such as phenols and minerals, had a much more significant increase.

## 5. Final Considerations and Future Perspectives 

Using chia oil directly is a promising alternative for application in products analogous to meat that aim to enhance the product’s tenderness, juiciness, and texture. An increase in nutritional value can be associated with this since chia oil has high omega-3 and omega-6 values, thereby providing a plant-based product with an excellent nutritional profile. To increase the amount of oil and add characteristics to products, such as extruded products (sausage, bologna, and ham) like meat, chia oil emulsions and nanoemulsions can be used.

As chia seeds, chia flour, and defatted chia flour have a high protein concentration and are an excellent source of valuable peptides, these can be important ingredients to increase the protein content of meat analogs and assist the technological properties. Furthermore, these products are alternative ingredients to those generally used as protein sources in meat analogs, such as soy, as there are consumers who do not like soy or are allergic to it. As in the bakery area, defatted chia flour is already used as an antioxidant ingredient to improve technological and sensory characteristics. Likewise, it presents itself as an excellent ingredient for meat-like products since the responses obtained for these products were positive. Furthermore, as the main antioxidant compounds present are not strongly affected by cooking the product, this suggests that the bioactive components present in defatted chia flour remain available during ingestion.

Chia mucilage has a positive effect when applied to many products, either as a fat substitute, technological aid, or substitute for gluten and phosphates. Chia mucilage can act in different functions to add quality and simulate meat products.

Aiming for sustainability and adding value to products, the synergistic use of different chia products is an innovative strategy, inserting the concept of biorefinery into the chia seed processing chain. Therefore, coproducts of chia mucilage and chia oil are promising ingredients.

## Data Availability

Data are contained within the article.
